# Cross-Reactive Human IgM-Derived Monoclonal Antibodies that Bind to HIV-1 Envelope Glycoproteins

**DOI:** 10.3390/v2020547

**Published:** 2010-02-04

**Authors:** Weizao Chen, Zhongyu Zhu, Huaxin Liao, Gerald V. Quinnan, Christopher C. Broder, Barton F. Haynes, Dimiter S. Dimitrov

**Affiliations:** 1 Protein Interactions Group, Center for Cancer Research Nanobiology Program, National Cancer Institute (NCI)-Frederick, National Institutes of Health (NIH), Frederick, MD 21702, USA; 2 Basic Research Program, Science Applications International Corporation-Frederick, Inc., NCI-Frederick, Frederick, MD 21702, USA; 3 Duke University Medical Center, Durham, NC 27710, USA; 4 Uniformed Services University of the Health Sciences, Bethesda, MD 20814, USA

**Keywords:** HIV-1, human, monoclonal antibody, IgM, gp120, immunogen

## Abstract

Elicitation of antibodies with potent and broad neutralizing activity against HIV by immunization remains a challenge. Several monoclonal antibodies (mAbs) isolated from humans with HIV-1 infection exhibit such activity but vaccine immunogens based on structures containing their epitopes have not been successful for their elicitation. All known broadly neutralizing mAbs (bnmAbs) are immunoglobulin (Ig) Gs (IgGs) and highly somatically hypermutated which could impede their elicitation. Ig Ms (IgMs) are on average significantly less divergent from germline antibodies and are relevant for the development of vaccine immunogens but are underexplored compared to IgGs. Here we describe the identification and characterization of several human IgM-derived mAbs against HIV-1 which were selected from a large phage-displayed naive human antibody library constructed from blood, lymph nodes and spleens of 59 healthy donors. These antibodies bound with high affinity to recombinant envelope glycoproteins (gp140s, Envs) of HIV-1 isolates from different clades. They enhanced or did not neutralize infection by some of the HIV-1 primary isolates using CCR5 as a coreceptor but neutralized all CXCR4 isolates tested although weakly. One of these antibodies with relatively low degree of somatic hypermutation was more extensively characterized. It bound to a highly conserved region partially overlapping with the coreceptor binding site and close to but not overlapping with the CD4 binding site. These results suggest the existence of conserved structures that could direct the immune response to non-neutralizing or even enhancing antibodies which may represent a strategy used by the virus to escape neutralizing immune responses. Further studies will show whether such a strategy plays a role in HIV infection of humans, how important that role could be, and what the mechanisms of infection enhancement are. The newly identified mAbs could be used as reagents to further characterize conserved non-neutralizing, weakly neutralizing or enhancing epitopes and modify or remove them from candidate vaccine immunogens.

## 1. Introduction

The ability of human immunodeficiency virus type 1 (HIV-1) to rapidly generate mutants and evade immune response is the major obstacle for development of protective, prophylactic HIV-1 vaccines. Therefore, candidate vaccine immunogens must be capable of eliciting broadly neutralizing antibodies (bnAbs) that inhibit viruses from different genetic subtypes. Several human monoclonal antibodies (hmAbs) such as b12 [1], X5 [2], 2G12 [3], 2F5 and 4E10 [4,5] exhibit potent and broad HIV-1 neutralizing activity *in vitro* and can prevent HIV-1 infection in animal models [6]. These bnAbs target structures on HIV-1 envelope glycoprotein (Env) that are crucial for virus-cell fusion. Therefore, Envs in various formats are potential candidate immunogens and have been evaluated in animal models and human clinical trials [7–10]. However, neutralization efficacy of the resulting sera as broad as that by those bnAbs has not been achieved by empirically using these Envs as immunogens [11]. It has been suggested that characterization of the epitopes of the bnAbs could help design vaccine immunogens that would be able to elicit these bnAbs or similar antibodies *in vivo* [12]. Although this approach is being vigorously pursued, none of the immunogens designed has yet efficiently elicited neutralizing antibodies with broad specificity.

IgM is the initial antibody that the host generates when an infectious agent or antigen is encountered. Activation of IgM-expressing B cells provides impetus for *in vivo* IgM-to-IgG isotype switch resulting in production of high-affinity neutralizing or non-neutralizing antibodies. It is therefore important to investigate the human IgM repertoire for HIV-1 Env-specific antibodies and understand how they interact with HIV-1 Envs and impact on viral infection which could help design effective immunogens. Previous attempts to select HIV-specific antibodies by use of non-immune libraries have resulted in antibodies with modest neutralizing activity and limited breadth of neutralization [13]. Studies by several groups show that human IgM antibodies play important roles not only in shaping humoral immunity against HIV-1 but also in inducing cell-mediated response because of their pentameric binding nature as well as their very efficient activity to activate complement. Torán *et al.* [14] indicated that *in vivo* IgM-to-IgG isotype switch and affinity maturation may be important for protection and long-term survival in certain HIV-1-infected individuals. Sheppard *et al*. [15] demonstrated that complement-deactivated serum from a healthy volunteer immunized with an HIV-1 clade C gp120 exhibited IgM-associated neutralizing activity that was significantly enhanced in the presence of fresh normal human serum as a source of complement. Bomsel *et al*. [16] showed that a human anti-HIV-1 gp41 IgM monoclonal antibody blocked the transcytotic route of HIV mucosal transmission *in vitro*.

In this study, we describe identification and characterization of several human IgM-derived mAbs selected from phage-displayed naive human antibody libraries constructed from healthy donors. These antibodies have high affinity and cross-reactivity with HIV-1 gp120s from different clades, and either neutralize or enhance weakly entry of virions pseudotyped with Envs from HIV-1 primary isolates. These data provide additional insights into the possible immune response that HIV-1 infection or viral Env-based immunization could elicit and help in the design of candidate vaccine immunogens that elicit potent neutralizers of early transmitted virus.

## 2. Results

### 2.1. Construction of a large naive human antibody library by using the phagemid vector pZYD-N1

A large (1.5 × 10^10^ members) phage-displayed naive human antibody antigen-binding fragment (Fab) library (designated m21) was constructed from peripheral blood B cells of 22 healthy donors, spleens of 3 healthy donors, and lymph nodes of 34 healthy donors as described in Materials and Methods. The phagemid used for library construction, pZYD-N1, was synthesized and briefly described previously [17]. To approximately estimate the sequence diversity of m21, 44 clones were randomly selected from the library and sequenced. No identical sequences of heavy and light chains were found; more than 80% of the clones are productive. Of the 44 clones 26 had kappa light chains and the other 18 - lambda light chains. M21 was panned against several HIV-1 Envs and human cancer-related antigens; significant enrichment was obtained with all the pannings (data not shown). These results indicate that the quality of the library is likely to be good.

### 2.2. Selection of HIV-1 Env-specific cross-reactive human IgM-derived antibodies

**Figure 1.**
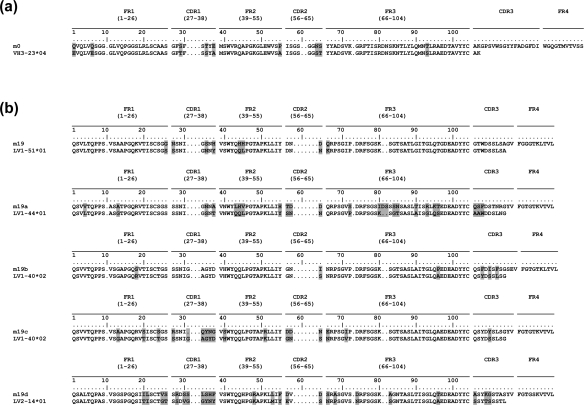
The amino acid sequences of heavy **(a)** and light **(b)** chains of the selected mAbs in alignment with the corresponding germlines of human antibody V genes. The CDRs and FRs are indicated according to the ImMunoGeneTics annotation (http://imgt.cines.fr/). The somatic mutations in the V genes of the selected antibodies are highlighted with gray background.

To identify human IgM-derived antibodies specific for HIV-1 Envs, we panned m21 against the gp140 of a clade B isolate, R2 (gp140_R2_). One of the selected antibody clones, R3H1, contained a TGA stop codon at the very beginning of the light chain due to a nucleotide deletion that resulted in reading-frame shift. The heavy chain variable domain (VH) of R3H1, designated m0, differs from the closest human germline sequence (VH3–23) by mutations distributed in all frameworks (FRs) and complementarity determining regions (CDRs) (Figure 1a). M0 contains 15 mutations in nucleotide sequence of the variable (V) region resulting in 9 mutations in amino acid sequence. We corrected the reading frame of the light chain by site-directed mutagenesis and the corrected antibody, designated R3H1m, was expressed at high levels in bacteria as an Fab. Fab R3H1m specifically bound with relatively high affinity to gp140_R2_ (EC_50_, ∼60 nM) and the gp140 of a clade C isolate, GXC44 (gp140_GXC44_) (EC_50_, ∼100 nM).

Because the antibody was initially selected as a functional heavy chain-only antibody, we hypothesized that the binding activity of R3H1m should be attributed mainly to the heavy chain of the antibody and the heavy chain could be paired with light chains derived from different germlines while retaining cross-reactivity with HIV-1 Envs. We cloned m0 as an isolated antibody domain and found that it was highly soluble, stable, monomeric, and expressed at high levels in bacteria [18]. In agreement with our hypothesis it specifically bound to gp140_R2_ although with low affinity (EC_50_, >1000 nM). We further constructed a light chain-shuffling Fab library (3 × 10^7^ members) based on the heavy chain of R3H1. To increase the probability for selection of cross-reactive antibodies the library was panned sequentially against gp140_R2_ and the gp140 of a clade F isolate, 14/00/4 (gp140_14/00/4_). Five unique clones were identified that contained exclusively lambda light chains (Figure 1b). They bound to gp140_R2_ and gp140_14/00/4_ with EC_50_ ranging from 2 to 80 nM (Table 1) while no significant binding to an unrelated antigen, bovine serum albumin (BSA), was observed (data not shown). One of the antibodies, m19, also bound with high affinity to the Envs of other three clade B isolates, gp120_Bal_, gp140_JRFL_ and gp140_Con-S_ [19]. These results suggest that m19 and possibly the other selected antibodies target conserved epitopes on gp120.

### 2.3. The selected antibodies did not neutralize, neutralized weakly or enhanced HIV-1 infections

**Figure 2.**
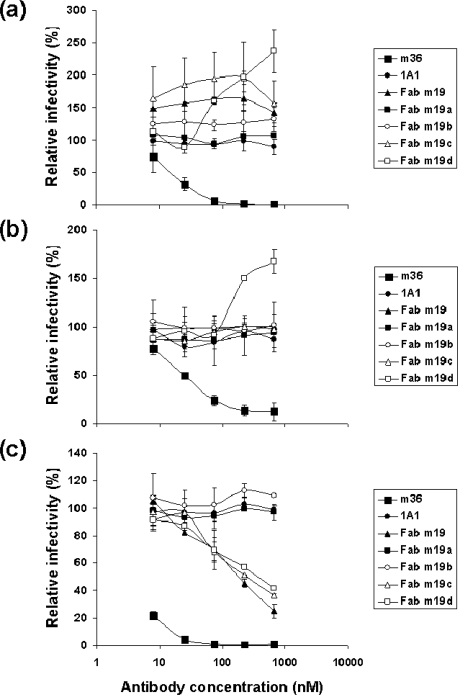
The *in vitro* activities of the selected mAbs against HIV-1 infection in a pseudovirus/cell line neutralization assay. The antibodies in Fab format were tested against two clade B primary R5 isolates, Bal **(a)** and JRFL **(b)**, and a clade B lab-adapted X4 isolate, IIIB **(c)**. A CD4i human antibody domain (domain antibody, dAb), m36, was used as a positive control and an irrelevant human dAb targeting the human insulin-like growth factor 2 (IGF-2), 1A1, was used as a negative control.

To test whether the five newly selected antibodies are capable of neutralizing HIV-1 primary isolates, we used viruses pseudotyped with Envs from HIV-1 isolates representing clades A, B and C and using either CCR5 (R5) or CXCR4 (X4) or both (R5X4) as a coreceptor. None of the antibodies exhibited potent broadly neutralizing activity. Four antibodies (Fabs m19, m19b, m19c and m19d) enhanced infection by Bal even at very low antibody concentration while a positive control antibody, m36 [17], showed efficient neutralization and a negative control antibody, 1A1, did not affect entry (Figure 2a); m19d enhanced infection by another clade B primary isolate, JRFL, at high concentrations (Figure 2b). Interestingly, the three antibodies that enhanced significantly infection by the Bal weakly neutralized IIIB which is a TCLA clade B X4 isolate (Figure 2c).

**Figure 3.**
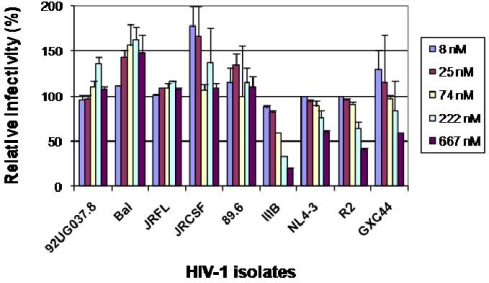
The activities of m19Fc against a small panel of HIV-1 isolates in a pseudovirus/cell line neutralization assay.

To find whether the activity of the antibodies is related to antibody size and how the viral infection could be affected by cross-linking of HIV-1 Envs, we generated a single-chain Fv fragment (scFv) (scFv m19) of m19 and a human IgG1 Fc-fusion protein (m19Fc) of scFv m19; m19 was selected for further characterization because its light chain was relatively less divergent from the germline (Figure 1b) and was the only one which did not contain any somatic mutations in the CDR3 of the light chain. When Bal was tested, the enhancing activity of scFv m19 was comparable to that of Fab m19 and slightly lower than that of m19Fc (data not shown). These data indicate that the antibody access to the epitope was not size-restricted and that the bivalency of the Fc fusion protein strengthened the antibody activity probably due to avidity effects. The ability of m19Fc to modulate HIV-1 infection *in vitro* was further tested with six additional clade B isolates and an isolate each from clade A and clade C, respectively (Figure 3). Enhancement was observed with JRCSF (clade B, R5) at very low m19Fc concentration and the enhancement was decreased with an increase in antibody concentration. M19Fc also conferred slight enhancement of infection by JRFL (clade B, R5) and 92UG037.8 (clade A, R5) at about 200 nM antibody concentration. It neutralized the clade B X4 isolates IIIB and NL4-3. It also neutralized R2, which is a clade B R5 isolate, displays some CD4 independence and is moderately neutralization sensitive, as well as GXC44 which is a clade C R5 isolate. No significant antibody activity was observed with the dual tropic clade B isolate 89.6. These results indicate that the antibody activity in terms of neutralization or enhancement varies when different isolates are tested and is not significantly correlated with the coreceptor usage and the CD4 dependency of the viruses in entry.

### 2.4. Characterization of the antibody epitopes

**Figure 4.**
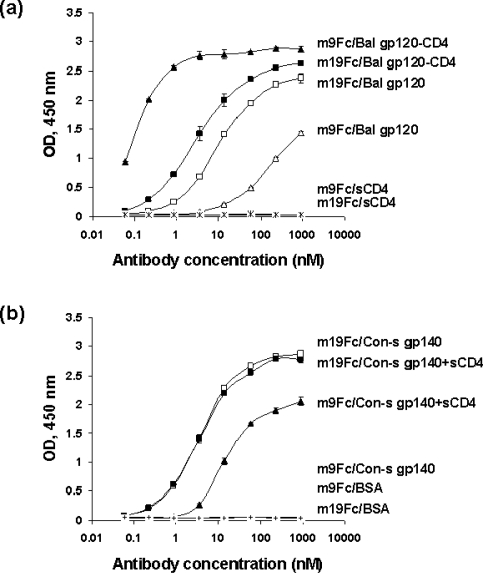
Binding of m19Fc to gp120_Bal_ **(a)** and gp140_Con-S_ **(b)** in the absence or presence of CD4. The Fc-fusion protein of a CD4i antibody, m9Fc, was used as a control.

**Figure 5.**
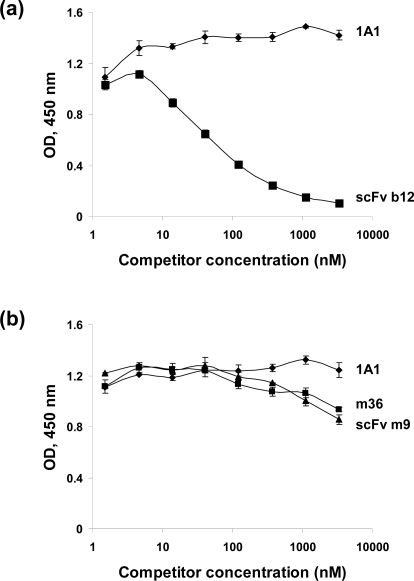
Competition of m19Fc with a CD4bs antibody, b12 **(a)**, and two CD4i antibodies, m36 and m9 **(b)**, in binding to gp120_Bal_ and to gp120_Bal_-CD4, respectively. An irrelevant human antibody, 1A1, was used as a negative control.

**Figure 6.**
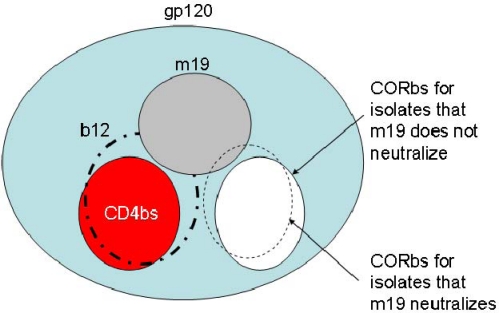
A schematic representation for the epitope (gray) of m19 on gp120 (blue). The red and white areas denote the CD4bs and the CORbs, respectively. The epitope (long dash-dotted circle on the left) of the CD4bs antibody, b12, is indicated in comparison with the CD4-binding area. The dashed circle on the right denotes the CORbs for some isolates that could partially overlap with the epitope of m19.

To approximately localize the antibody epitopes and begin to elucidate the underlying mechanisms of antibody neutralizing or enhancing activity, we measured the antibody binding to Envs from different isolates alone and in complex with CD4 as well as the antibody competition with well-characterized antibodies. M19Fc bound to a single-chain polypeptide analogue of the HIV-1 gp120-CD4 complex (gp120_Bal_-CD4) [20] with threefold higher affinity (EC50, ∼2.5 nM) than to gp120_Bal_ (EC50, ∼10 nM) alone as measured by an ELISA (Figure 4a). It did not react with sCD4 suggesting that the antibody targeted a structure on gp120 that was outside the CD4-binding site (CD4bs) and could undergo CD4-induced (CD4i) conformational changes. The conformational changes on gp120_Bal_ after CD4 binding were confirmed by a CD4i bnAb, m9Fc [21], which exhibited dramatically higher binding to gp120_Bal_-CD4 than to gp120_Bal_ (Figure 4a). To find out whether the antibody bound to the conventional CD4i epitopes, we used gp140_Con-S_, which was a synthetic Env designed by aligning the consensus Env sequences of group M [19]. This Env, by design, is composed by shorter V1, V2, V4 and V5 loops and therefore, might expose regions around the variable loops that might contain conserved neutralizing determinants. However, m9 epitope as a representative for CD4i epitopes is still completely hidden on gp140_Con-S_ because m9Fc did not bind in the absence of sCD4, it did bind with high affinity in the presence of sCD4 (Figure 4b). However, m19Fc showed comparable binding to gp140_Con-S_ with or without sCD4 (Figure 4b) suggesting that the antibody does not precisely target CD4i epitopes but other structures on the gp120. Although m19Fc was not directed against the CD4-binding pocket, it strongly competed with the CD4bs bnAb, scFv b12, which has binding surface larger than that of CD4 [22] (Figure 5a). M19Fc also competed although weakly with two CD4i antibodies, scFv m9 and m36 [17] (Figure 5b). These results suggest that the epitope of m19Fc is in very close proximity to the CD4bs and the coreceptor-binding site (CORbs) which overlaps with CD4i epitopes on gp120 (Figure 6).

### 2.5. Somatic mutations are required for the antibody binding activity

**Figure 7.**
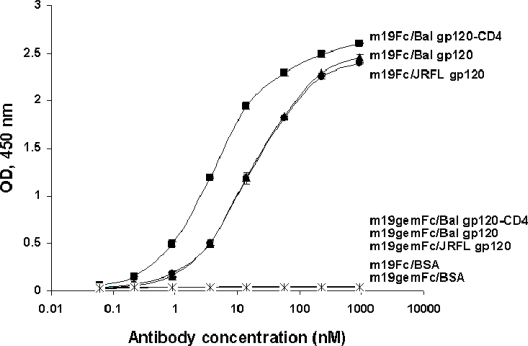
Binding of the germline-like antibody of m19 to different gp120s in the absence or presence of CD4. The original antibody, m19Fc, was used as a positive control.

To estimate the possibility of rapid elicitation of these antibodies *in vivo* when an HIV-1 gp120-related immunogen is administered, we generated a germline-like scFv of m19 (scFv m19gem) and measured its binding to Envs from different isolates in the presence or absence of sCD4. No obvious interaction was observed with scFv m19gem when several Envs that bound the original m19 were tested (data not shown). Previous studies showed that the binding activity of an antibody in the form of Fab or scFv could be increased up to thousands of times when it is converted to dimeric formats such as an IgG. We therefore fused scFv m19gem to the Fc portion of a human IgG1 to gain possible avidity effects. Still, the Fc-fusion protein of scFv m19gem (m19gemFc) did not show measurable binding to the Envs with or without sCD4 (Figure 7). These results suggest that significant somatic diversification is required for the m19 corresponding germline antibody to achieve recognition of the m19 epitope on gp120. They also indicate that in some individuals certain IgM antibodies could undergo somatic hypermutations to relatively high-affinity binders to the Env in the absence of HIV-1 infection or immunization with Env.

## 3. Discussion

Vaccines based on recombinant Envs have failed to prevent viral infection in human efficacy trials likely because of their inability to elicit neutralizing antibodies that are broad enough to combat the extremely high variability of the virus. Recent success was reported but it is modest and needs to be repeated and further examined [23]. To date only several bnAbs have been isolated from HIV-1 patients suggesting difficulties to elicit such antibodies *in vivo*. However, the presence of epitopes recognized by these bnAbs on Envs argues that they are still a potential template for vaccine design. Different strategies aiming at improving the presentation of neutralizing epitopes are rigorously pursued. Analysis of the structures of Envs shows that most of their surface is hidden from humoral immune responses by glycosylation and oligomeric occlusion [24,25]. The CD4bs and the CORbs on gp120 are also flanked by loop structures which may further limit the access by antibodies generated by the human immune system [24,25]. Therefore, one strategy focuses on the use of modified Envs, in which the variable loops have been deleted [26–29] or glycosylation removed [30,31] or both [32] in order to increase the exposure of the neutralizing epitopes. Based on the finding that the neutralizing capacity of the antibodies is associated with their ability to bind to native trimeric Envs on the virus but does not correlate with binding to isolated monomeric Envs, another strategy focuses on preservation or re-construction of the functional trimeric Envs [33,34]. The essential concept is that immunizing with a close mimic of the functional trimer will improve the chances of eliciting neutralizing antibodies. Yet another strategy is to use fusion intermediates formed during virus entry [35,36] or their mimics [37]. HIV-1 entry is initiated by binding of viral gp120 with the receptor CD4 and a coreceptor on the target cell surface. These interactions lead to intermediate Env conformations that may include conserved structures useful for vaccine design. However, elicitation of desirable level and breath of neutralizing antibodies with the immunogens generated based on these strategies has not been achieved. While modification of Envs based solely on the analysis of the interaction with neutralizing antibodies is not sufficient to create effective vaccines, we propose that the inherent capacity of the human immune system in response to HIV-1 infection should be explored. We have hypothesized that investigation of human IgM repertoire for HIV-1 Env-specific antibodies and understanding how they react with Envs, evolve and impact on viral infection would help design effective HIV-1 immunogens.

It has been previously found that IgM-based response confers substantial effects on HIV-1 infection which are especially relevant in the context of acute infection [14–16]. In this study, we describe the identification and characterization of several high-affinity human IgM-derived mAbs against HIV-1 from phage-displayed naive human antibody libraries constructed from healthy donors. Although their potency *in vitro* appears minor the roles they could play in mediating HIV-1 infection *in vivo* should not be underestimated. IgM antibodies are highly effective in activating complement system to destroy the virus or infected cells. The antibodies selected in this study target highly conserved regions on gp120 which are in very close proximity to the CD4bs and the CORbs. Because these two regions on gp120 are vital for virus entry and harbor neutralizing epitopes, HIV-1 has evolved strategies such as steric occlusion to protect it from humoral immunity [24,25]. Here we suggest that the possible high immunogenicity of the conserved non-neutralizing epitopes of these antibodies could further divert the immune system from responses to neutralizing epitopes. Recent studies [38,39] showed that cross-reactive non-neutralizing antibodies (IgMs and IgGs) were elicited by immunizing mice with recombinant HIV-1 gp140s suggesting the same possibility in humans. Because the antibodies we selected are less divergent (totally about 15 amino acid mutations in the V gene products for the heavy and light chains) (Figure 1) than the known bnAbs which all contain extensive somatic mutations (on average >30 mutations) compared to germline antibodies (data not shown), they could be more easily elicited. In addition, these antibodies could directly block the access of some neutralizing antibodies generated by the human immune system, especially those targeting the CD4bs or CORbs. This was supported by our finding that m19Fc partially reversed the neutralization by a CD4i antibody, m36 [17] (data not shown). These results further support previous propositions to reduce the immunogenicity of unwanted epitopes when gp120/gp140 is used as an immunogen.

We found that the closest germline-like antibody to m19 does not bind an Env (Figure 7). We observed similar lack of binding of germline-like b12, 2F5 and 2G12 to Envs [40–42]. Moreover, we found that there was a lack of or no high-affinity binders to Envs in the germline-like antibody repertoire of the human cord blood whereas high-affinity antibodies against other human infectious agents including SARS coronavirus and henipaviruses could be easily selected (Weizao Chen, Emily Streaker and Dimiter S. Dimitrov, in preparation). The identified antibodies against SARS coronavirus protein S exhibited nanomolar affinity and were very close to germline in sequence. By analyzing the previously identified antibody sequences we found that all of the cross-reactive neutralizing antibodies against HIV-1 were highly diversified from their germline sequences; in contrast, the antibodies against SARS coronavirus and henipaviruses had only limited number of mutations [40–42]. These results indicate that extensive somatic mutations could be required for high-affinity binding to conserved HIV-1 Env structures. One could speculate that the maturation pathways for some HIV-1 antibodies are initiated by immunogens that are different from the Envs; such immunogens could be used in combination with Env-based immunogens to guide the immune system for elicitation of potent bnAbs by additional somatic hypermutation and selection. This is in line with our proposition to elicit bnAbs by using one or more primary immunogens that are different than Envs but can lead to intermediates on the maturation pathways that can be subsequently further somatically hypermutated to matured bnAbs [40–42].

Except for the conventional antigen-antibody interaction, gp120s also bind to a small population of human antibodies containing products of mainly VH3 gene family and exhibit superantigen properties by activating the human B cells expressing such antibodies on cellular membrane [43]. The core motif of the superantigen-binding site (SBS) on gp120 is a discontinuous structure spanning the V4 variable domain and the amino-terminal region flanking the C4 constant domain [44]. The determinants on the antibody VH domains critical for gp120 binding are not clear yet while the putative important regions were identified in the FR1 and 3; a modeling study showed that most of the potential contact residues were located on the face of the VHs opposite to the interface for interaction with light chain; other positions in the CDR1 and 2 also influenced the binding [45]. Moreover, the gp120-positive antibody VH genes showed >96% similarity to the germlines suggesting that the somatic hypermutations may have adverse effects on gp120 binding [45]. The antibodies described in this study contained VH3-23 gene products (Figure 1a) and therefore, their high binding could be due to the superantigen-like interactions. However, our results showed that the isolated VH of these antibodies, m0, bound to gp120s with affinity (EC_50_, >1000 nM) much lower than that (EC_50_, 2–80 nM) of the Fabs. In addition, the epitope of one (m19) of these antibodies was mapped to a structure in very close proximity to the CD4bs and the CORbs on gp120 but not to the defined SBS. These results suggest that the majority, if not all, of the binding activities of the selected antibodies should be contributed by the conventional antigen-antibody recognition.

One should note that these antibodies may not represent the native ones because they were selected from phage libraries where the heavy and light chains of the antibodies are randomly recombined. However, previous studies [46–48] have shown that selection of high-affinity antibodies from such libraries results in antibodies which are identical or very similar to those occurring in the host from which the libraries are made. It has been shown that at least in some systems the antigen selected antibodies such as the autoantibodies against thyroid peroxidase (TPO) from large random phage libraries have the same pairings as those from small libraries where the cognate pairing is preserved and that pairing between light and heavy chain is not promiscuous [49,50]. The study by Chapal *et al*. [48] directly demonstrates that antibodies derived from combinatorial libraries are likely to represent *in vivo* pairings, leading to high affinity antibody fragments mimicking the binding of serum autoantibodies to TPO. De Wildt *et al*. [51] analyzed the cognate pairings of heavy and light chain variable domains in 365 human IgG+ B cells from peripheral blood and established that the pairings are largely random. The antibodies we selected were originated from large families of genes – heavy chain from VH3 and light chain from VL1 (Figure 1). Importantly, the pairings for two of them, m19 and m19d, have been found in the very limited number (365) of truly human antibodies previously analyzed [51]. Moreover, some (e.g., m19 and m19b) of these antibodies are relatively close to their corresponding germlines (Figure 1). These data suggest the possibility of eliciting such antibodies *in vivo* during HIV-1 infection or gp120-based immunization.

## 4. Materials and Methods

### 4.1. Cells, viruses, plasmids, gp120, gp140, and antibodies

We purchased the 293T cells from ATCC. Other cell lines and plasmids used for expression of various HIV-1 Envs were obtained from the National Institutes of Health AIDS Research and Reference Reagent Program (ARRRP). Recombinant gp140s were produced in our laboratories. Gp120_Bal_ and the single-chain fusion protein gp120_Bal_-CD4 were kindly provided by T. Fouts (Institute of Human Virology, Baltimore; currently at Profectus, Baltimore, MD). Horseradish peroxidase (HRP)-conjugated anti-FLAG tag antibody and HRP-conjugated anti-human IgG (Fc-specific) antibody were purchased from Sigma-Aldrich (St. Louis).

### 4.2. Library construction

M21 was constructed by using phagemid pZYD-N1 according to the reported protocols [52]. The sources for amplification of antibody gene fragments are commercially purchased poly A+ RNAs (BD Biosciences, San Jose, CA) from peripheral blood B cells of 22 healthy donors, spleens of three donors and lymph nodes of 34 healthy donors, respectively.

### 4.3. Selection of antibodies against HIV-1 antigens

M2l was used for selection of antibodies against HIV-1 antigens conjugated to magnetic beads (Dynabeads M-270 epoxy; DYNAL Inc., New Hyde Park, NY) as described previously [53]. 5, 5, 2.5 and 1 μg of gp140_R2_ were used in the first, second, third and fourth round of panning, respectively. Clones that specifically bound to gp140_R2_ were identified from the third and fourth round by using monoclonal phage ELISA (mpELISA) as described [53].

A light chain-shuffling Fab library (3 × 10^7^ members) was further constructed based on the heavy chain of R3H1. The light chain repertoire was also harvested from a naive human Fab library (5 × 10^9^ members) constructed from peripheral blood B cells of 10 healthy donors [53], in addition to that from m21. The new library was panned sequentially with two gp140s from different clades. For the sequential panning, 5 and 2.5 μg of gp140_R2_ were used in the first and third round, respectively; antigens were alternated with 5 and 2.5 μg of gp140_14/00/4_ during the second and fourth round, respectively. Clones that bound to both gp140_R2_ and gp140_14/00/4_ were identified from the fourth round by using mpELISA as described [53].

### 4.4. Construction of the scFv and Fc-fusion proteins of selected antibodies

The following primers were used: m36F, 5′-TGG TTT CGC TAC CGT GGC CCA GCC GGC CCA GGT GCA GCT GGTG-3′ (sense); m19FcR: 5′-GTG AGT TTT GTC GGG CCC TAG GAC GGT CAG CTT GG-3′ (antisense).

For construction of scFv, the full-length original or germline scFv gene fragment was synthesized (GenScript, Piscataway, NJ), digested with SfiI and cloned into pComb3X. To generate Fc-fusion protein, the scFv gene was PCR (primers m36F and m19FcR) amplified by using the synthetic scFv gene fragment as a template. The new scFv products appended with SfiI and ApaI restriction sites on both sides were digested and cloned into pSecTagB-Fc.

### 4.5. Expression and purification of antibodies

The scFv and Fab were expressed in *E. coli* HB2151, as described previously [53]. The bacterial pellet was collected after centrifugation at 5,000 × *g* for 10 min and resuspended in PBS (pH 7.4) containing 0.5 million-unit polymixin B (Sigma-Aldrich). After 30 min incubation with rotation at 50 rpm at room temperature, it was centrifuged at 25,000 × *g* for 25 min at 4 °C. The supernatant was used for purification of scFv and Fab by immobilized metal ion affinity chromatography (IMAC) by using Ni-NTA resin (Qiagen, Valencia, CA) according to manufacturer’s protocols. Fc-fusion proteins were expressed in 293 free style cells. 293Fectin (Invitrogen, Carlsbad, CA) was used to transfect 293 free style cells according to the instructions of the manufacturer. Three days post-transfection, the culture supernatant was harvested and used for purification by using nProtein A Sepharose 4 Fast Flow (GE Healthcarezcomx, Piscataway, NJ).

### 4.6. ELISA

Binding and competition ELISAs were performed as described previously [17].

### 4.7. Pseudovirus neutralization assay

Viruses pseudotyped with HIV-1 Envs were prepared by cotransfection of 70–80% confluent 293T cells with pNL4–3.luc.E-R- and pSV7d constructs encoding HIV-1 Envs by using the PolyFect transfection reagent (Qiagen) according to manufacturer’s instruction. Pseudotyped viruses were obtained after 48 h by centrifugation and filtration of cell culture through 0.45 μm filters. For neutralization, viruses were mixed with different concentrations of antibodies for 1 h at 37 °C, and then the mixture was added to about 1.5 × 10^4^ HOS-CD4-CCR5 (used for all R5 and dual tropic viruses) or HOS-CD4-CXCR4 cells grown in each well of 96-well plates. Luminesence was measured after 48 h by using the Bright-Glo Luciferase Assay System (Promega, Madison, WI) and a LumiCount microplate luminometer (Turner Designs). Mean relative light units (RLU) for duplicate wells were determined. Relative infectivity (%) was calculated by the following formula: (average RLU of antibody-containing wells/average RLU of virus-only wells) × 100.

## 5. Conclusions

A major finding of this study is the identification and characterization of several HIV-1-specific human IgM-derived mAbs and their highly conserved epitopes. Such antibodies were rarely investigated and reported. Further characterization of these antibodies, their dynamics and epitopes could provide knowledge that in addition to its usefulness for basic understanding of immune responses to HIV-1 could also help in the design of candidate vaccine immunogens that elicit potent neutralizers of early transmitted virus.

## Figures and Tables

**Table 1.** Binding (EC_50_, nM) of antibodies to Envs from different clades.

Antibodies	Envs (clade)				
	gp140_R2_ (B)	gp120_Bal_ (B)	gp140_JRFL_ (B)	gp140_Con-s_ (B)	gp140_14/00/4_ (F)
Fab m19	20	130	120	120	10
Fab m19a	24	ND	ND	ND	24
Fab m19b	8	ND	ND	ND	6
Fab m19c	74	ND	ND	ND	2
Fab m19d	24	ND	ND	ND	10

ND, not done
